# Supplementary material for: The interactome of intact mitochondria by cross-linking mass spectrometry provides evidence for coexisting respiratory supercomplexes
Source: Mol Cell Proteomics. 2017 Dec 8;17(2):216–32. doi: 10.1074/mcp.RA117.000470 (PMC5795388; doi:10.1074/mcp.RA117.000470)
Supplement: Supplemental Data [file supp_RA117.000470_133922_0_supp_23978_fzffwf.zip › spectra_annotation/mito_spectra_annotation/493-2-18-1-1-1.pdf]

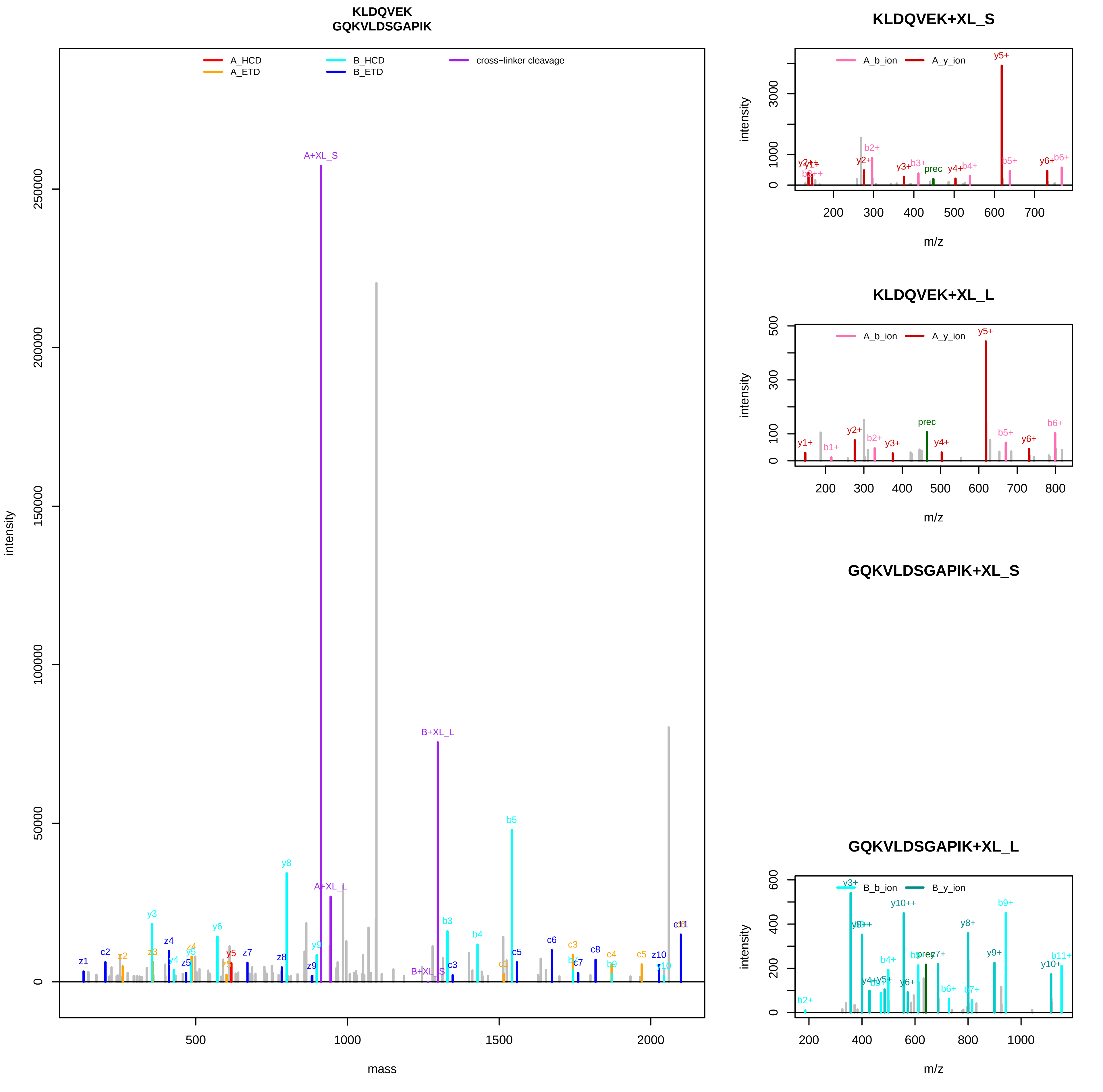
KLDQVEK
GQKVLDSGAPIK
A_HCD
A_ETD
B_HCD
B_ETD
cross−linker cleavage
A+XL_S
B+XL_L
A+XL_L
B+XL_S
intensity
mass
0
50000
100000
150000
200000
250000
500
1000
1500
2000
z1 c2 z2 z3 y3 z4 y4 z5 y5 z4 c5 y5 y6 z7 z8 y8 z9 y9 b3 c3 b4 c1 b5 c5 c6 c3 b7 c7 c8 c4 b9 c5 z10 y10 c11
KLDQVEK+XL_S
A_b_ion
A_y_ion
y5+ b2+ y2+ y3+ b3+ prec y4+ b4+ b5+ y6+ b6+
intensity
m/z
0
1000
3000
200
300
400
500
600
700
KLDQVEK+XL_L
A_b_ion
A_y_ion
y5+ y1+ b1+ y2+ b2+ y3+ prec y4+ b5+ y6+ b6+
intensity
m/z
0
100
300
500
200
300
400
500
600
700
800
GQKVLDSGAPIK+XL_S
GQKVLDSGAPIK+XL_L
B_b_ion
B_y_ion
y3+ y10++ b9+ y8+ b4+ y9+ b11+ y10+ b2+ y6+ b6+ b7+ prec y7+
intensity
m/z
0
200
400
600
200
400
600
800
1000
